# Methylene blue can act as an antidote to pesticide poisoning of bumble bee mitochondria

**DOI:** 10.1038/s41598-021-94231-3

**Published:** 2021-07-19

**Authors:** Mikhail Syromyatnikov, Ekaterina Nesterova, Tatiana Smirnova, Vasily Popov

**Affiliations:** 1 Department of Genetics, Cytology and Bioengineering, Voronezh State University, Voronezh, Russia 394018; 2 Laboratory of Metagenomics and Food Biotechnology, Voronezh State University of Engineering Technolgies, Voronezh, Russia 394036

**Keywords:** Bioenergetics, Energy metabolism

## Abstract

The population of bumble bees and other pollinators has considerably declined worldwide, probably, due to the toxic effect of pesticides used in agriculture. Inexpensive and available antidotes can be one of the solutions for the problem of pesticide toxicity for pollinators. We studied the properties of the thiazine dye Methylene blue (MB) as an antidote against the toxic action of pesticides in the bumble bee mitochondria and found that MB stimulated mitochondrial respiration mediated by Complex I of the electron transport chain (ETC) and increased respiration of the mitochondria treated with mitochondria-targeted (chlorfenapyr, hydramethylnon, pyridaben, tolfenpyrad, and fenazaquin) and non-mitochondrial (deltamethrin, metribuzin, and penconazole) pesticides. MB also restored the mitochondrial membrane potential dissipated by the pesticides affecting the ETC. The mechanism of MB action is most probably related to its ability to shunt electron flow in the mitochondrial ETC.

## Introduction

A decline in the population of pollinators is a serious worldwide problem^1–5^ that has become a question of humankind biological safety. Bumble bees are among the most important pollinators^6^ that are also commonly used in agriculture (*Bombus impatiens*, *Bombus ignitus*, and *Bombus terrestris*)^7^. The populations of bumble bees have considerably declined in the North America, Europe, and other world regions^8–10^, presumably because of the toxic effects of pesticides^11–14^. The reasons for the loss of pollinating insects include habitat destruction^15–17^, various pathogens, e.g., such as viruses^18,19^ and *Varroa* mites^20–22^, climate change^23–25^, reduced food supply^26–28^, and global use of pesticides^21,23,26,27,29,30^. The synergistic effect of pesticides and pathogens may be the a major factor in the global decline of pollinators^30,31^. Among pesticides neonicotinoids are often blamed for the loss of bees^32,33^, whereas fungicides can promote the effect of these pesticides on pollinators^34–37^. Pesticides affect insect reproduction, behavior, and development of bumble bee colonies^38^.Thus, pesticides are believed to the most significant deleterious factor in the colony collapse disorder^39^.

Some pesticides target mitochondria and reduce the efficiency of oxidative phosphorylation, e.g., by inhibiting the electron transport chain (ETC) complexes. For example, insecticide hydramethylnon inhibits the activity of Complex 3^40,41^. Insecticides/acaricides fenazaquin^24^, tolfenpyrad, and pyridaben^42,43^ inhibit Complex 1. Some pesticides, e.g., pro-insecticide chlorfenapyr, uncouple oxidative phosphorylation in the mitochondria, which reduces the efficiency of this process and leads to the energy loss in the cells^44^.

Many non-mitochondrial pesticides can also affect the activity of mitochondria. Fungicide Pristine ® inhibits mitochondrial function in honeybees^45^. Fipronil and imidacloprid influence multiple functional parameters of bee mitochondria and reduce the activity of these organelles^46^. Organophosphate pesticides often cause oxidative stress and mitochondrial dysfunction^47^. Pyrethroids can impair various mitochondrial functions, disrupt formation of the mitochondrial membrane potential, increase production of reactive oxygen species, alter the fluidity of the mitochondrial membrane lipids, and cause the damage of mitochondrial DNA^48^. Organochloride pesticides also can damage mitochondria^49^. For example, thiacloprid alters transcription of genes associated with oxidative phosphorylation^50^. Some fungicides inhibit mitochondrial respiration and uncouple oxidative phosphorylation in the bumble bee flight muscles^51^.

At the same time, improving mitochondrial functions can reduce the toxic effect of non-mitochondrial pesticides on bumble bees^52^. Hence, the use of inexpensive and available antidotes against pesticides can be one of the solutions for the problem of pesticide toxicity in pollinators, first of all, bumble bees and honey bees. However, the number of such antidotes is very limited. It was found that glucocorticoids and cyclophosphamide significantly alleviate the toxic effects of paraquat, an efficient herbicide used worldwid^53^. Sucralfate^54^ and ellagic acid^55^ can also prevent the toxicity of paraquat. Lysine acetylsalicylate significantly decreases paraquat toxicity in mammals^56^.

Atropine alleviates pesticide poisoning^57–60^, while oximes act as antidotes against specific pesticides^59,61–63^. Magnesium sulfate can be used for managing the poisoning with organophosphorus pesticides^64^ and aluminum phosphide^65^. Pralidoxime and vitamin K are antidotes of organophosphorus insecticides and anticoagulant rodenticides, respectively^66^. 1,8-Naphthalic anhydride is a potential antidote against fungicides^67^. Ozone, both in its gaseous form and dissolved in water, can be used to remove difenoconazole and linuron from carrots^68^.

Methylene blue (MB) is a thiazine dye that has recently attracted a significant attention of researchers because of its newly discovered biological activities. It was found that MB has an antidote effect in methemoglobinemia^69,70^and poisoning with carbon monoxide and cyanide^71,72^. In the mitochondria, MB plays an important role due to its activity as a catalytic redox cycler^73^ and can serve as an alternative electron acceptor^74^. MB was found to improve mitochondrial respiration and to decrease oxidative stress in the hearts of diabetic rats^75^, as well as to maintain the function and structure of the retina treated with rotenone (Complex 1 inhibitor)^76^. It also inhibits multiple amine oxidases, thereby preventing chloroacetaldehyde formation. Taking into account the above properties of MB, we believe that MB can be used as an antidote to a wide range of pesticides affecting animals, including pollinators. An important advantage of MB is that it can be added to the syrup fed to bumble bees.

Here, we studied the properties of MB as a potential antidote against the toxic effects of pesticides. To evaluate the protective effect of MB, we measured mitochondrial respiration and membrane potential of the bumble bee mitochondria subjected to the action of various pesticides and treated with MB.

## Materials and methods

### Bumble bees

*B. terrestris* (L.) males were provided by the Technology of Bumble Bee Rearing Ltd. (Voronezh, Russia). The bumble bees were kept in cylindrical cages (diameter, 14 cm; height, 7 cm) in the dark at 27–28.5 °C at the air humidity of 55–68%. The bumblebees were fed with 60% inverted sugar syrup.

### Pesticides

Mitochondrial pesticides chlorfenapyr (CAS Number 122453-73-0), hydramethylnon (CAS Number 67485-29-4), pyridaben (CAS Number 96489-71-3), tolfenpyrad (CAS Number 129558-76-5), and fenazaquin (CAS Number 120928-09-8) and non-mitochondrial pesticides **i**midacloprid (CAS Number 138261-41-3), deltamethrin (CAS Number 52918-63-5), malathion (CAS Number 121-75-5), metribuzin (CAS Number 21087-64-9), penconazole (CAS Number 66246-88-6), cypermethrin (CAS Number 52315-07-8), and esfenvalerate (CAS Number 66230-04-4) were from Sigma-Aldrich, USA. All pesticides were dissolved in dimethyl sulfoxide (DMSO) at a concentration of 10 mM.

### Isolation of mitochondria

Bumble bee mitochondria were isolated as described earlier^77^. For each individual experiment, nine *B. terrestris* males were frozen at − 18 °C for 15 min. The thoraces were separated from the heads and abdomens, placed in 12 ml of ice-cold isolation medium (220 mM mannitol, 100 mM sucrose, 1 mM EGTA, 2 mg/ml fat-free BSA, 20 mM HEPES, pH 7.4) and disintegrated with a 15-ml Dounce tissue grinder. All procedures were performed at 0–4 °C. The homogenate was centrifuged for 5 min at 600 g, and the supernatant was centrifuged for 10 min at 10,000 g. The resulting pellet was resuspended in the washing medium (isolation medium without BSA) and centrifuged for 10 min at 10,000 g. The pellet was resuspended in 100 μl of the washing medium and kept on ice. Isolated mitochondria were used in the experiments within 2 h after isolation. Protein content in the mitochondria was determined with the BCA assay kit (Pierce Biotechnology, USA).

### Mitochondrial respiration

The oxygen consumption rate (OCR) in the isolated mitochondria was measured by the amperometric method with a Clark oxygen electrode (Hansatech Instruments, USA). All measurements were performed at 24 °C in 1 ml of incubation medium containing 220 mM mannitol, 100 mM sucrose, 1 mM EGTA, 4 mM potassium phosphate, 20 mM HEPES (pH 7.4), and 5 mM respiratory substrate. MB was added to the concentration of 2 μM (MB concentration was chosen based on the earlier studies of the MB effect on the rat and mouse mitochondria^78,79^. The pesticides were directly added to the oxygraph chamber. The pesticide concentration in the oxygraph chamber was chosen to produce the maximum inhibitory effect (as previously determined for the mitochondrial respiration in vitro, unpublished data) and varied depending on the pesticide (Table 1). The effect of MB and each of the pesticides on mitochondrial respiration was measured in 6 repetitions (n = 6).

**Table 1:** Concentration of pesticides in the oxygraph chamber/cuvette.

Pesticide	Type	Concentration, μM
Chlorfenapyr	Insecticide/mitochondrial	30
Hydramethylnon	Insecticide/mitochondrial	30
Pyridaben	Insecticide/mitochondrial	10
Tolfenpyrad	Insecticide/mitochondrial	10
Fenazaquin	Insecticide/mitochondrial	10
**I**midacloprid	Insecticide/non-mitochondrial	60
Deltamethrin	Insecticide/non-mitochondrial	40
Malathion	Insecticide/non-mitochondrial	60
Metribuzin	Herbicide/non-mitochondrial	40
Penconazole	Fungicide/non-mitochondrial	40
Cypermethrin	Insecticide/non-mitochondrial	40
Esfenvalerate	Insecticide/non-mitochondrial	40

### Membrane potential measurements

The membrane potential of the isolated mitochondria was evaluated from changes in the fluorescence of the membrane potential probe Safranin O using a Hitachi F-7000 spectrofluorometer (Hitachi, Japan)^80^ at the excitation wavelength of 495 nm and emission wavelength of 586 nm. Incubation medium (1 ml) contained 220 mM mannitol, 100 mM sucrose, 1 mM EGTA, 4 mM potassium phosphate, 0.2 mg/ml BSA, 20 mM HEPES (pH 7.4), 100–120 μg of mitochondrial protein, 2–4 nmol Safranin O, and 10 mM  respiratory substrate. MB was added to the concentration of 2 μM. The pesticides were added directly to the cuvette; the pesticide concentration was the same as in the assessment of mitochondrial respiration (Table 1). The effect of MB and each of the pesticides on mitochondrial membrane potential was measured in 6 repetitions (n = 6).

### Hydrogen peroxide production by the mitochondria

Was measured with the Amplex Red Ultra dye (Sigma, CШA) as described early^81^ using a Hitachi F-7000 spectrofluorimeter in 1 ml of the incubation medium (see above) containing 2 μM Amplex Red Ultra, 100–200 μg of mitochondria, and 1 mg/ml horseradish peroxidase (excitation wavelength, 568 nm; emission wavelength, 581 nm). MB was added directly to the cuvette to the concentration of 2 μM. The pesticide concentration was the same as in the assessment of mitochondrial respiration (Table 1). The effect of MB and each of the pesticides on mitochondrial hydrogen peroxide production was measured in 6 repetitions (n = 6).

### Statistical analysis

Was performed with the STATISTICA software (StatSoft Inc., Tulsa, OK, USA). The results were expressed as mean ± SD. The differences were analyzed with ANOVA and were considered significant at *p* < 0.05. The effect of MB and each of the pesticides was measured in 6 repetitions (*n* = 6).

## Results

### Effect of mitochondria-targeted pesticides and MB on the mitochondria

The respiration of mitochondria from the bumble bee flight muscles on various respiratory substrates (malate, pyruvate, glutamate, proline, succinate, α-glycerophosphate) was measured in the presence and absence of MB. We found that MB stimulated mitochondrial respiration mediated by Complex I on the following respiratory substrates: pyruvate, malate, pyruvate + malate, pyruvate + proline, pyruvate + glutamate. The highest respiratory rate (in the presence of ADP) was observed on pyruvate + glutamate (respiratory control, 6.1); however, the highest respiratory control (14.1) was observed on pyruvate. Thus, the respiratory rate on pyruvate in the absence of MB was 91.51 ± 5.2 nmol O_2_/min mg protein and increased to 115.30 ± 7.21 nmol O_2_/min mg protein after MB addition. At the same time, MB failed to stimulate mitochondrial respiration on succinate and α-glycerophosphate.

We also studied the effect of MB on the respiration of mitochondria treated with the mitochondria-targeted pesticides chlorfenapyr, hydramethylnon, pyridaben, tolfenpyrad, and fenazaquin (Sigma-Aldrich, USA).

**Figure 1:**
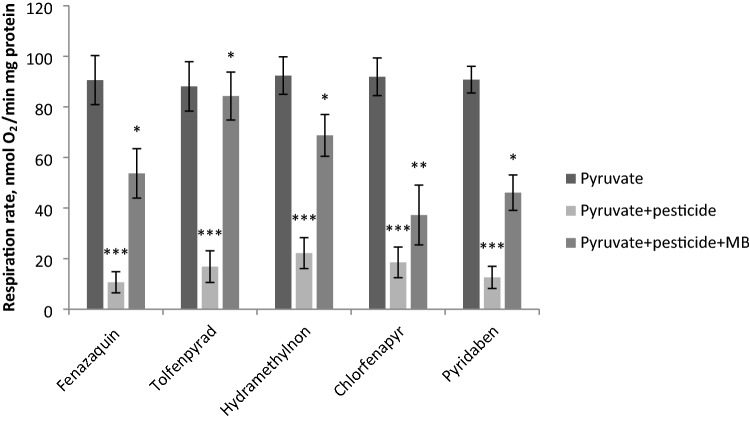
Respiration of mitochondria on pyruvate (ETC complex I substrate) in the presence of mitochondria-targeted pesticides and after MB addition (the data are shown as mean ± SD, *n* = 6). Pesticides, MB and mitochondria (120 μg) were directly added to the oxygraph chamber. MB was added to the concentration of 2 μM. The concentration of the pesticides in oxygraph chamber see in Table 1. * Statistically significant differences in the mitochondrial respiration rate in the presence of pesticide and pesticide + MB, p < 0.001. ** Statistically significant differences in the mitochondrial respiration rate in the presence of pesticide and pesticide + MB, p < 0.01. *** Statistically significant differences in the mitochondrial respiration rate in the absence and presence of pesticide, p < 0.001.

All mitochondria-targeted pesticides inhibited respiration mediated by Complex I (Fig. 1), which was then restored by the addition of MB.

Both MB and pesticides produced a statistically significant effect on the mitochondrial respiration: fenazaquin F(2, 15) = 138.82, p < 0.001; tolfenpyrad F(2, 15) = 128.68, p < 0.001; hydramethylnon F(2, 15) = 142.52, p < 0.001; chlorfenapyr F(2, 15) = 142.30, p < 0.001; pyridaben F(2, 15) = 289.15, p < 0.001. The most pronounced inhibitory effect was observed for fenazaquin (from 90.6 ± 9.7 to 10.7 ± 4.2 nmol O_2_/min mg protein) and pyridaben (from 90.8 ± 5.3 to 12.6 ± 4.4 nmol O_2_/min mg protein) (Tukey's test, p < 0.001). Tolfenpyrad, hydramethylnon, and chlorfenapyr suppressed respiration mediated by Complex I to a lesser extent (Tukey's test, p < 0.001). MB activated pesticide-inhibited mitochondrial respiration on pyruvate. Thus, 2 μM MB increased the respiration rate inhibited by fenazaquin from 10.7 ± 4.2 to 53.7 ± 9.8 nmol O_2_/min mg protein (Tukey's test, p < 0.001). Addition of MB to the mitochondria inhibited by tolfenpyrad increased oxygen consumption from 16.8 ± 6.3 to 84.3 ± 9.5 nmol O_2_/min mg protein (Tukey's test, p < 0.001). In the cases of pyridaben and hydramethylnon, MB increased the respiration rate from 12.6 ± 4.4 to 46.1 ± 7.0 nmol O_2_/min mg protein and from 22.2 ± 6.1 to 68.7 ± 8.3 nmol O_2_/min mg protein, respectively (Tukey's test, p < 0.001). MB addition to the mitochondria inhibited with chlorfenapyr, increased the respiration rate from 18.5 ± 5.6 to 37.2 ± 10.0 nmol O_2_/min mg protein (Tukey's test, p < 0.01). Therefore, MB restored mitochondrial respiration suppressed by the pesticides inhibiting Complex 1 of the ETC.

**Figure 2:**
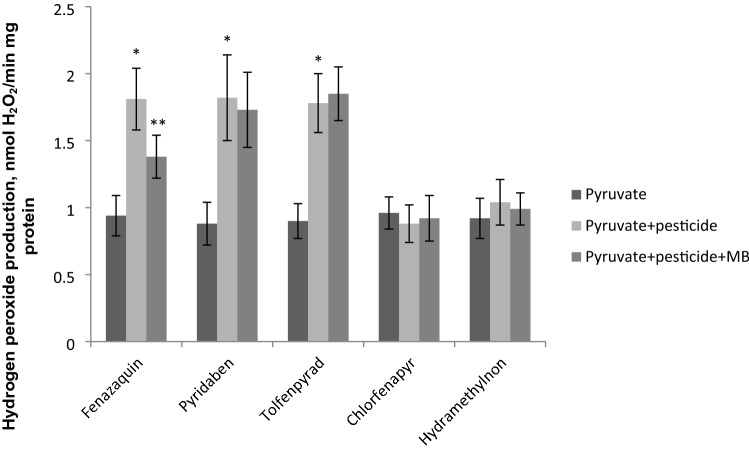
Hydrogen peroxide production of mitochondria on pyruvate (ETC complex I substrate) in the presence of mitochondria-targeted pesticides and after MB addition (the data are shown as mean ± SD, *n* = 6). Pesticides, MB and mitochondria were directly added to the cuvette. MB was added to the concentration of 2 μM. The concentration of the pesticides in cuvette see in Table 1. * Statistically significant differences in the hydrogen peroxide production of mitochondria in the absence and presence of pesticide, p < 0.001. ** Statistically significant differences in the hydrogen peroxide production of mitochondria in the presence of pesticide and pesticide + MB, p < 0.01.

Next, we estimated the effect of MB and pesticides on the generation of reactive oxygen species (ROS) by the flight muscle mitochondria in vitro. The rate of ROS production was measured on two substrates: pyruvate (respiration mediated by Complex I) and α-glycerophosphate (respiration mediated by the mitochondrial α-glycerophosphate dehydrogenase). We found that MB did not affect the rate of ROS generation by the mitochondria on pyruvate; the production of hydrogen peroxide in the presence MB was 0.9 ± 0.08 nmol H_2_O_2_/min mg protein vs. 0.8 ± 0.10 nmol H_2_O_2_/min mg protein in the absence of MB. Fenazaquin (F(2, 15) = 33.39, p < 0.001), tolfenpyrad (F(2, 15) = 23.39, p < 0.001) and pyridaben (F(2, 15) = 47.31, p < 0.001) have increased the production of hydrogen peroxide on the pyruvate (Fig. 2). No differences in the H_2_O_2_ production before and after MB and pesticide addition were found in the mitochondria on α-glycerophosphate (3.1 ± 0.27 H_2_O_2_/min mg protein in the absence of MB vs. 3.5 ± 0.32 H_2_O_2_/min mg protein in the presence of MB).

After fenazaquin MB reduced the production of hydrogen peroxide by mitochondria from 1,81 ± 0,23 to 1,38 ± 0,16 nmol H_2_O_2_/min mg protein (Tukey's test, p < 0.01). MB did not reduce or increase production of hydrogen peroxide by mitochondria after other pesticides.

**Figure 3:**
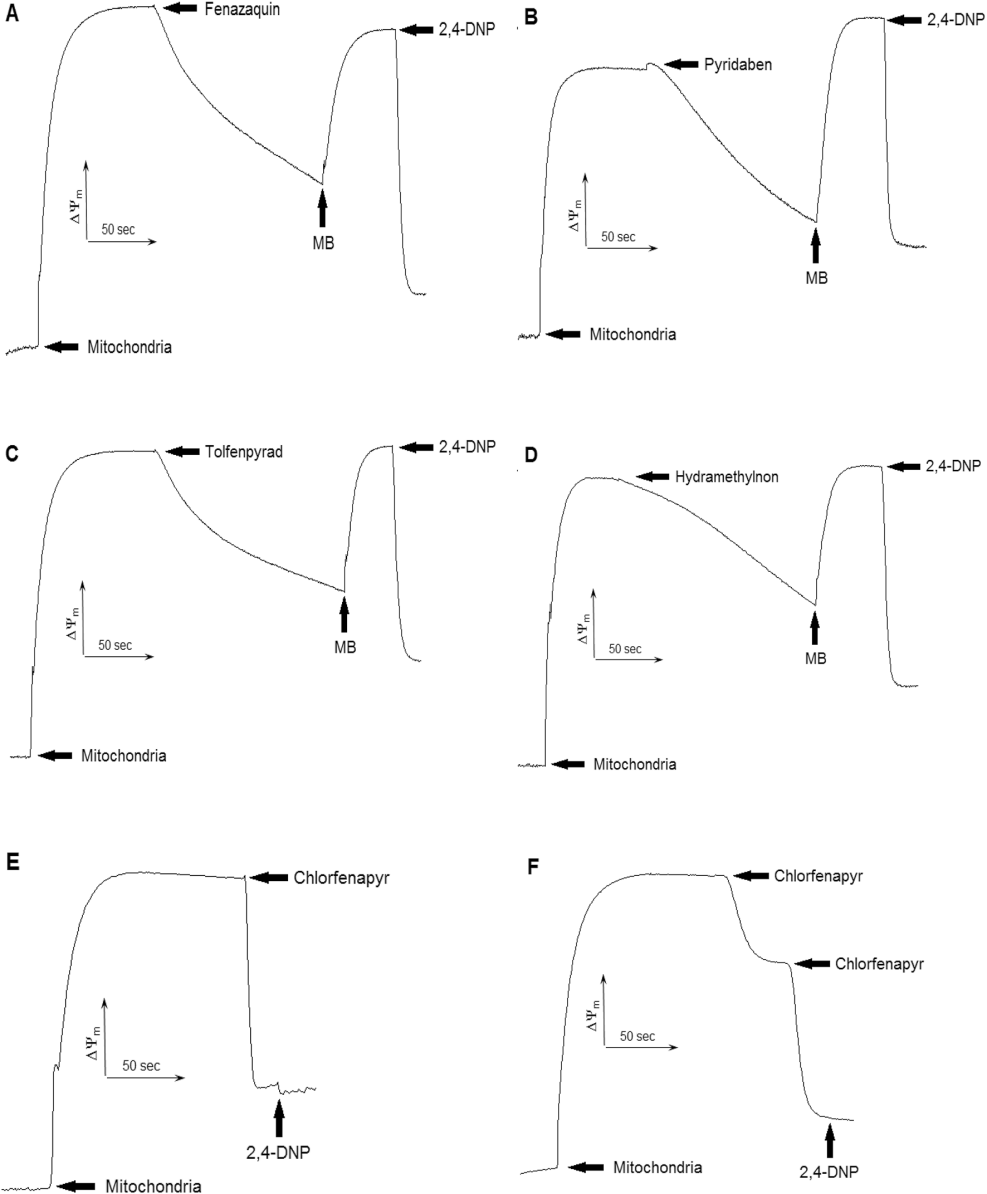
MB restored mitochondrial membrane potential on glutamate + pyruvate (ETC complex I substrate) after dissipation by fenazaquin (**A**), pyridaben (**B**), tolfenpyrad (**C**), and hydramethylnon (**D**). Chlorfenapyr uncoupled mitochondrial respiration on both glutamate + pyruvate (**E**) and α-glycerophosphate (**F**); 2,4-DNP, 2,4-dinitrophenol. Pesticides, MB and mitochondria were directly added to the cuvette. MB was added to the concentration of 2 μM. The concentration of the pesticide in cuvette see in Table 1, *n* = 6.

MB restored the mitochondrial membrane potential dissipated by the pesticides affecting the ETC (Fig. 3).

Therefore, MB was able to restore both mitochondrial respiration and mitochondrial membrane potential necessary for the ATP production in the mitochondria. We also found that chlorfenapyr uncoupled mitochondrial respiration, since its addition to the mitochondria oxidizing α-glycerophosphate (Fig. 3F) led to the complete loss of membrane potential.

### Effect of non-mitochondrial pesticides and MB on the mitochondria

**Figure 4:**
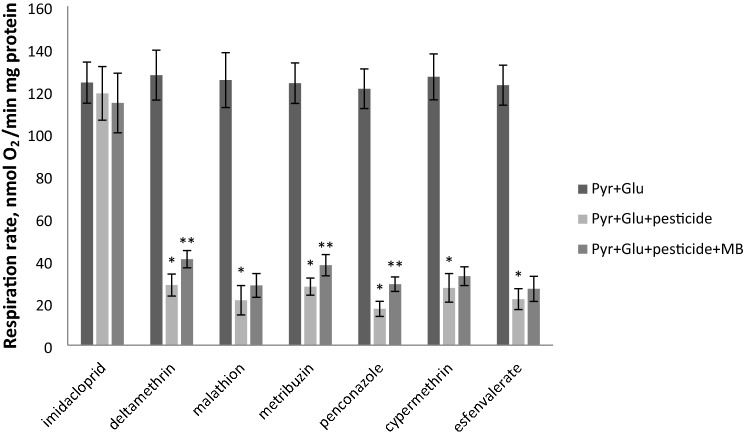
Effect of pesticides and MB on the mitochondrial respiration on ETC complex I substrates (the data are shown as mean ± SD, *n* = 6); *Pyr* pyruvate, *Glu* glutamate. Pesticides, MB and mitochondria (120 μg) were directly added to the oxygraph chamber. MB was added to the concentration of 2 μM. The concentration of the pesticide in oxygraph chamber see in Table 1, *n* = 6. *Statistically significant differences in the mitochondrial respiration rate in the absence and presence of pesticide, p < 0.001. **Statistically significant differences in the mitochondrial respiration rate in the presence of pesticide and pesticide + MB, p < 0.05.

We also evaluated the respiration rate of the isolated flight muscle mitochondria on glutamate + pyruvate (respiration mediated by Complex I) in the presence of ADP after incubation with the pesticides imidacloprid, deltamethrin, malathion, metribuzin, penconazole, cypermethrin, and esfenvalerate and following addition of MB. The components of the reaction mixture were added to the oxygraph cell in the following order: mitochondria, ADP, pesticide, MB. The effect of the pesticides and MB on the mitochondrial respiration is shown in Fig. 4.

The effect of pesticides (except imidacloprid) and MB on the mitochondrial respiration was statistically significant: deltamethrin F(2, 15) = 288.77, p < 0.001; malathion F(2, 15) = 244.90, p < 0.001; metribuzin F(2, 15) = 377.70, p < 0.001; penconazole F(2, 15) = 517.61, p < 0.001; cypermethrin F(2, 15) = 311.68, p < 0.001; esfenvalerate F(2, 15) = 392.78, p < 0.001. Deltamethrin noticeably (from 127.5 ± 11.8 to 28.4 ± 5.2 nmol O_2_/min mg protein) inhibited mitochondrial respiration (Tukey's test, p < 0.001); subsequent addition of MB stimulated respiration to 40.6 ± 4.1 nmol O_2_/min mg protein (Tukey's test, p < 0.05). Metribuzin inhibited respiration from 123.7 ± 9.6 to 27.6 ± 4.0 nmol O_2_/min mg protein (Tukey's test, p < 0.001); MB increased respiration to 37.7 ± 5.0 nmol O_2_/min mg protein (Tukey's test, p < 0.05). Penconazole inhibited respiration from 121.0 ± 9.4 to 17.1 ± 3.6 nmol O_2_/min mg protein (Tukey's test, p < 0.001), while MB stimulated respiration to 28.8 ± 3.4 nmol O_2_/min mg protein (Tukey's test, p < 0.05). Cypermethrin and esfenvalerate suppressed mitochondrial respiration from 126.7 ± 10.8 to 27.0 ± 6.8 nmol O_2_/min mg protein (Tukey's test, p < 0.001) and from 122.7 ± 9.5 to 21.7 ± 4.9 nmol O_2_/min mg protein (Tukey's test, p < 0.001), respectively; in both cases, the following addition of MB failed to increase the respiration rate. Hence, MB partially restored mitochondrial respiration suppressed by deltamethrin, metribuzin and penconazole (pesticides affecting Complex 1). No differences in the H_2_O_2_ production before and after non-mitochondrial pesticide addition were found in the mitochondria.

## Discussion

Here, we demonstrated that MB stimulates respiration mediated by Complex I of the ETC in the mitochondria from the bumble bee flight muscles. Note that no stimulation of mitochondrial respiration was observed on α-glycerophosphate or succinate, as respiration on these two substrates is not mediated by Complex I. Presumably, MB acts as a redox component of the ETC due to its ability to participate in the NADH oxidation by Complex I and to transfer electrons on cytochrome *c*, thus providing an alternative electron transfer in the mitochondria.

MB addition caused no statistically significant changes in the rates of ROS production by the mitochondria on α-glycerophosphate and pyruvate, which might be explained by the presumed MB ability to shunt electrons in Complex I, thus ensuring partial reduction of the electron flow through NADH dehydrogenase, which contributes most to the ROS generation.

MB restored respiration in the mitochondria treated with the mitochondria-targeted pesticides, such as pyridaben, chlorfenapyr, fenazaquin, tolfenpyrad, and hydramethylnon. As reported earlier, chlorfenapyr disturbs oxidative phosphorylation in the mitochondria^44^ (uncouples oxidative phosphorylation and suppresses ATP production), which might result in the organism death. According to our data, chlorfenapyr inhibited Complex I in the bumble bee flight muscle mitochondria. It also increased the mitochondrial respiration rate on α-glycerophosphate (unpublished data), which was most probably due to the uncoupling effect of this compound. These data confirm our hypothesis that chlorfenapyr uncouples oxidative phosphorylation, as well as inhibits Complex I in the bumble bee flight muscle mitochondria.

Hydramethylnon is known to suppress the activity of Complex III of the mitochondrial ETC^41^. We found that that this pesticide indeed inhibited Complex III, because it decreased (1.3 times) the respiration rate on both pyruvate and α-glycerophosphate. However, inhibition by hydramethylnon was more pronounced for the respiration on pyruvate (see above), which suggests that this pesticide inhibited both Complex I and Complex III in the flight muscle mitochondria.

The mechanism of MB action as an antidote might be related to the specific properties of this compound. MB has been long known as an electron carrier^82,83^. It is also a redox mediator capable of oxidizing intramitochondrial NADH and transferring electrons to the downstream components of the ETC. This effect was termed “alternative electron transport”^84^. MB can be reduced by NADH, FADH_2_, and α-glycerophosphate to leucomethylene blue (MBH2), which is then oxidized primarily by cytochrome *c*^85^. This suggests that MB donates electrons to the Qo ubiquinol-binding site of Complex III.

We have shown earlier that a wide range of pesticides, including non-mitochondrial ones, negatively affect the bioenergetic parameters of mitochondria from the bumble bee flight muscles^86^. Interestingly, MB also stimulated respiration after treatment of the mitochondria with the non-mitochondrial pesticides, such as deltamethrin, metribuzin and penconazole, although to a lesser extent than after the treatment with the mitochondria-targeted pesticides. It is possible that MB can act as an antidote against other (non-mitochondrial) pesticides, but this hypothesis requires further investigation.

The mechanism of the MB-mediated stimulation of respiration in the mitochondria from the bumble bee flight muscles after exposure to pesticides remains unclear. Most probably, the activity of MB is related to its ability to shunt electrons in the mitochondrial ETC. Although further studies are required for the comprehensive understanding of the MB action mechanisms, the obtained results already suggest that MB can be used as an antidote against the toxic action of pesticides in pollinators.

## Conclusions

MB stimulated respiration mediated by Complex I in the bumble bee flight muscles mitochondria and restored respiration in the mitochondria treated with the mitochondria-targeted pesticides, such as pyridaben, chlorfenapyr, fenazaquin, tolfenpyrad, and hydramethylnon. MB also stimulated respiration in the mitochondria subjected to the action of non-mitochondrial pesticides, such as deltamethrin, metribuzin and penconazole, although to a lesser extent than in the mitochondria treated with the mitochondria-targeted pesticides. MB restored the mitochondrial membrane potential dissipated by the pesticides affecting the ETC. Taken together, these data demonstrate that MB can be used to reduce the toxicity of pesticides in pollinators. For instance, MB can be added to the syrup used for feeding bumble bees, which might be convenient for the insect treatment in both indoor (greenhouses) and outdoor environments. However, further studies on the effects of MB on bumble bees and other pollinators are needed to elucidate the precise mechanism of action of this antidote.
